# Secondary metabolites constituents and antioxidant, anticancer and antibacterial activities of *Etlingera elatior* (Jack) R.M.Sm grown in different locations of Malaysia

**DOI:** 10.1186/s12906-015-0838-6

**Published:** 2015-09-23

**Authors:** Ali Ghasemzadeh, Hawa Z. E. Jaafar, Asmah Rahmat, Sadegh Ashkani

**Affiliations:** Department of Crop Science, Faculty of Agriculture, Universiti Putra Malaysia, 43400 Serdang, Selangor Malaysia; Department of Nutrition & Dietetics, Faculty of Medicine & Health Sciences, Universiti Putra Malaysia, 43400 UPM Serdang, Selangor Malaysia; Institute of Tropical Agriculture, Universiti Putra Malaysia, 43400 Serdang, Selangor Malaysia; Department of Agronomy and Plant Breeding, Shahr-e- Rey Branch, Islamic Azad University, Tehran, Iran

**Keywords:** Etlingera elatior, DPPH, FRAP, MCF-7, MDA-MB-231, Antibacterial activity

## Abstract

**Background:**

*Etlingera elatior* is a well-known herb in Malaysia with various pharmaceutical properties.

**Methods:**

*E. elatior* flowers grown in three different locations of Malaysia (Kelantan, Pahang and Johor), were investigated for differences in their content of secondary metabolites (total phenolics [TPC], total flavonoids [TFC], and total tannin content [TTC]) as well as for their antioxidant, anticancer, and antibacterial properties. Phenolic acids and flavonoids were isolated and identified using ultra-high performance liquid chromatography (UHPLC). Ferric reducing antioxidant potential (FRAP) and 1,1-diphenyl-2-picrylhydrazyl (DPPH) assays were used to evaluate the antioxidant activities. The anticancer activity of extracts was evaluated using the MTT (3-(4,5-dimethylthiazol-2-yl)-2,5-diphenyltetrazolium bromide) assay.

**Results:**

When extracted with various solvents (aqueous and ethanolic), samples from the different locations yielded significantly different results for TPC, TFC, and TTC as well as antioxidant activity. Aqueous extracts of *E. elatior* flowers collected from Kelantan exhibited the highest values: TPC (618.9 mg/100 g DM), TFC (354.2 mg/100 g DM), TTC (129.5 mg/100 g DM), DPPH (76.4 %), and FRAP (6.88 mM of Fe (II)/g) activity with a half-maximal inhibitory concentration (IC_50_) of 34.5 μg/mL compared with extracts of flowers collected from the other two locations. The most important phenolic compounds isolated in this study, based on concentration, were: gallic acid > caffeic acid > tannic acid > chlorogenic acid; and the most important flavonoids were: quercetin > apigenin > kaempferol > luteolin > myricetin. Extracts of flowers from Kelantan exhibited potent anticancer activity with a IC_50_of 173.1 and 196.2 μg/mL against the tumor cell lines MCF-7 and MDA-MB-231 respectively, compared with extracts from Pahang (IC_50_ = 204.5 and 246.2 μg/mL) and Johor samples (IC_50_ = 277.1 and 296.7 μg/mL). Extracts of *E. elatior* flowers also showed antibacterial activities against *Staphylococcus aureus, Bacillus subtilis, Listeria monocytogenes, Escherichia coli, Salmonella typhimurium,* and *Pseudomonas aeruginosa* with minimal inhibitory concentrations (MIC) ranging from 30 to >100 μg/mL.

**Conclusions:**

In general, therefore, based on the potent antioxidant and anticancer activity of flower extracts, it appears that *E. elatior* grown in the North-east of Malaysia (Kelantan) is a potential source of therapeutic compounds with anti-cancer activity.

## Background

**Fig. 1:**
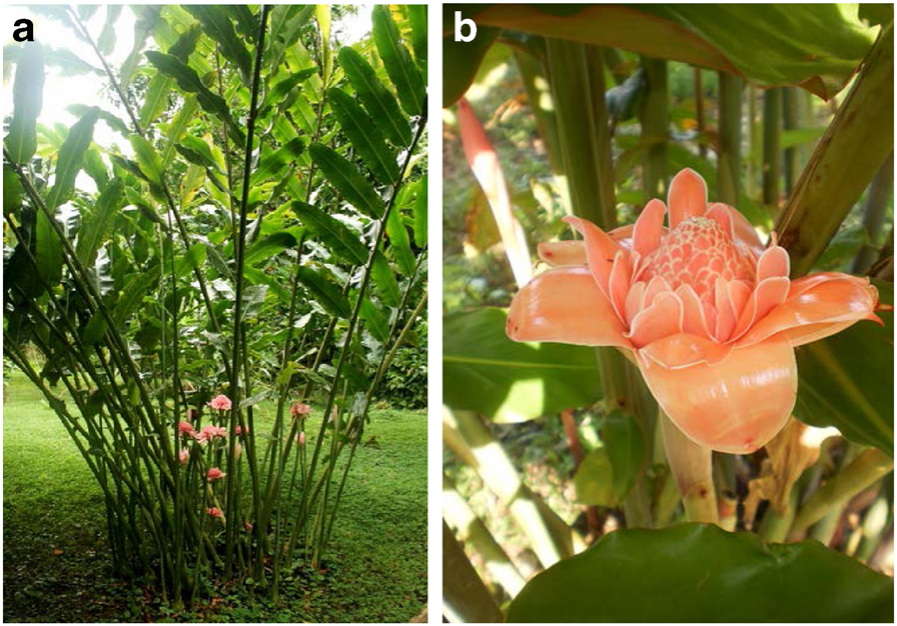
*Etlingera elatior* (Jack) R.M.Sm.; **a** whole plant, **b** flower

Herbs and natural products are important sources of medicinal compounds and their beneficial healing effects have been recognized since ancient times. The characteristics and therapeutic effects of natural bioactive compounds, especially from plant sources including herbs and spices, have been investigated extensively. Phytochemicals are important compounds found in medicinal plants which provide health benefits for humans further than those attributed to macronutrients and micronutrients [1]. According to a report by the World Health Organization, 80 % of the population in developing countries depends on traditional medicine for their primary health care, and 85 % of traditional medicine is derived from plant extracts [2]. In Malaysia, herbs and spices are generally consumed raw and fresh similar to vegetables (salad), especially by the Malay community. *Etlingera elatior* (Jack) R.M.Sm. (Fig. 1) locally known in Malaysia as Kantan, Bunga kantan, Bunga siantan, and Torch ginger in English, is a plant that belongs to the ginger family (*Zingiberaceae*). *E. elatior* is commonly found in South Asia where it is traditionally used to treat earache and clean wounds [3, 4] and as a spice in Malaysian dishes including, *Penang laksa*, *nasi kerabu,* and *nasi ulam* [5, 6]. The young shoots and flower buds of the plant are consumed raw by indigenous communities in Malaysia and Thailand. In addition, *E. elatior* has been reported to have various other properties including antioxidant [7, 8], anticancer [9] anti-proliferative [10], antibacterial [4] and cytotoxic activity [9]. The pharmacological activity of herbs is correlated to their content of phytochemicals. Various phytochemical groups and constituents have been identified in *E. elatior*. Methanol (80 %) extracts of dried flowers of *E. elatior* contain flavonoids (quercetin and kaempfrol), terpenoids, saponins, tannins, and carbohydrates [11]. Methanol (50 and 100 %) extracts of dried flowers of *E. elatior* contain tannin and anthocyanin respectively [7]. Commonly, water and organic solvents (methanol, ethanol, acetone, or diethyl ether) have been used for extraction of phytochemicals and bioactive compounds from herbs and spices and the extraction methods and solvent type affect the percent recovery of the various materials [12, 13]. Different herbs and spices have very variable compositions of phytochemicals and bioactive compounds so that it is very difficult to predict the optimal conditions for extracting individual plant materials. Therefore, it is important to refer to the sampling location and environmental parameters when considering the content of phytochemicals and the beneficial effect on health exerted by herbs. Some plants of Malaysian origin have been screened for their pharmaceutical activity, yet little is known about their constituents that may contribute to their medicinal functionality. This information is necessary to validate the safety, traditional uses, and to standardize preparations of these plants. To the best of our knowledge, there is little information on the content and pharmaceutical quality of flavonoid and phenolic compounds in Malaysian *E. elatior.* Additionally, it is not known whether the production of secondary metabolites differs between *E. elatior* grown in different geographical locations in Malaysia. This study aimed to characterize the phytochemical content and to investigate the antioxidant, anticancer and antibacterial activity of *E. elatior* flowers grown in three different areas [North-east (Kelantan), Central (Pahang), and South-east (Johor)] of Malaysia.

## Methods

### Plant sampling 

Samples of *E. elatior* flowers were collected from three different areas of Malaysia: Kelantan (North-east), Pahang (Central), and Johor (South-east). The samples were identified by Mr. Thiyagu Devarajan from the Malaysian Agriculture Research and Development Institute (MARDI) as *E. elatior* . Flowers were harvested, washed with distilled water and shade dried. After drying process, samples were stored at −20 °C for future analysis.

### Extraction

Dried flowers (50 g) were ground into powder followed by extraction with distilled water and ethanol (1 L). Solutions were refluxed for 2 h at 65 °C, then cooled, and filtered through Whatman filter paper (No. 1) in a filter funnel. This was followed by evaporation under reduced pressure in an Eyela rotary evaporator to remove excess solvent. The residue was freeze-dried and dried extracts were kept at −20 °C for future analysis.

### Total phenolic content

Extracts of flowers (200 μL) were diluted in 20 mL of distilled water. Folin-Ciocalteu reagent (10-fold diluted; 1 mL) was added and the mixture was incubated in total darkness for 10 min at room temperature. Sodium carbonate 7.5 % (1 mL) was then added and incubated for 30 min, then the absorbance of the solution was read at 765 nm using a spectrophotometer (UV2550, Shimadzu, Japan) [14]. Different concentrations of gallic acid were used to prepare a calibration curve.

### Total flavonoid content

Flower extracts (1 mL) were mixed with NaNO_2_ in a methanolic solution (4 mL, 1:5, w/v) and incubated at room temperature for 6 min. Then, 0.3 mL of AlCl_3_ solution (1:10, w/v) was added, the reagents were mixed well, and the reaction was allowed to stand for another 6 min. Immediately after that, 1 M NaOH solution (2.0 mL) was added to each extract and incubated for 10 min at room temperature. The absorbance of the solutions was read at 510 nm using a spectrophotometer (UV2550, Shimadzu, Japan) [15]. Different concentrations of quercetin standard were used to prepare a calibration curve.

### Total tannin content

Total tannins content were determined according to the method of Morrison et al. [16] with some modification. 0.5 mL of extract was diluted with methanol to made up to 5 mL. Extract was mixed with 25 mL of vanillin reagent (1 g vanillin in 100 mL methanol) and 25 mL of 4 % HCl in methanol. The mixture was kept for 15 min at room temperature in dark place, and absorption was measured at 500 nm using a spectrophotometer (UV2550, Shimadzu, Japan). Methanol was used as a blank. All samples were analyzed in triplicate.

### Separation and analysis of flavonoids and phenolic acids

Ultra-high performance liquid chromatography (UHPLC, 1290 Infinity Quaternary LC System, Agilent, Santa Clara, CA, USA) was used to separate and identify the phenolic acids. The chromatographic system conditions were set as follows: mobile phase, 0.03 M orthophosphoric acid (A) and methanol HPLC grade (B); detector, UV 280 nm; column, C18 column (5.0 μm, 4.6 mm inner diameter [ID] × 250 mm); column oven temperature, 35 °C; and flow rate, 1.0 mL/min. Gradient elution was performed as follows: 0 min 40 % B, 10 min 100 % B, 15 min 100 % B, and 20 min 40 % B. Linear regression equations were calculated using Y = aX ± b, where X is the concentration of the related compound and Y the peak area of the compound obtained from UHPLC. The linearity was established by the coefficient of determination (R^2^).

### *vitro* evaluation of antioxidant activity

#### 1,1-Diphenyl-2-picrylhydrazyl (DPPH) assay

The DPPH assay was used in order to evaluate the free radical scavenging activity of *E. elatior* extracts. DPPH was dissolved in methanol at a concentration of 100 μM. The DPPH solution (3 mL) was mixed with 3 mL of various concentrations (10, 20, 40, 80, and 160 μg/mL) of *E. elatior* extracts and incubated in a dark room for 20 min at 27 °C. After incubation, the absorbance of the samples was read at 517 nm using a spectrophotometer (UV2550, Shimadzu, Japan). Butylhydroxytoluene (BHT) and α-tocopherol were used as positive controls [17]. The scavenging activity was calculated using the following formula:

1 $$ \%\ \mathrm{inhibition}=\left(\frac{\left[\mathsf{absorba}\mathrm{n}\mathsf{c}{\mathrm{e}}_{\mathsf{co}\mathrm{n}\mathsf{trol}}\hbox{--}\ \mathsf{absorba}\mathrm{n}\mathsf{c}{\mathrm{e}}_{\mathsf{sampl}\mathrm{e}}\right]}{\left[\mathsf{absorba}\mathrm{n}\mathsf{c}{\mathrm{e}}_{\mathsf{co}\mathrm{n}\mathsf{trol}}\right]}\right)\mathrm{x}\kern0.5em 100 $$

### Ferric Reducing Antioxidant Potential (FRAP) Assay

The stock solutions consisted of 10 volumes of 300 mM acetate buffer (pH = 3.6), 1 volume of 10 mM TPTZ (2,4,6-tripyridyl-S-triazine) solution in 40 mM HCl, and 1 volume of 20 mM FeCl_3_ solution. Acetate buffer (25 mL) and TPTZ (2.5 mL) were mixed (FRAP solution), and 2.5 mL FeCl_3_ was added. Flower extracts (100 μL) and deionized water (300 μL) were added to 3 mL of the FRAP solution. Solution was mixed well and incubated for 30 min in a water bath (at 37 °C). The absorbance of the resultant solution was measured at 593 nm using a spectrophotometer (U-2001, Hitachi Instruments Inc., Tokyo, Japan) with acetate buffer used as the blank. A standard curve was prepared using various concentrations of FeSO_4_ × 7H_2_O. The value for the blank absorbance was subtracted from that of the sample and used to calculate the FRAP value [18].

### Determination of Anticancer Activity

#### Cell Culture and Treatment

Human breast carcinoma cell lines (MCF-7 and MDA-MB-231) and normal human mammary epithelial cells (MCF-10A) were cultured in RPMI 1640 media (Roswell Park Memorial Institute) containing 10 % fetal bovine serum (FBS). Cell lines were incubated overnight at 37 °C in 5 % CO_2_ to allow the cells to attach to the culture plates.

MTT (3-(4,5-Dimethylthiazol-2-yl)-2,5-diphenyltetrazolium bromide) Assay

The assay was conducted as follows: Cells were seeded in 96-well plates at a density of 1 × 10^4^ cells/well in 100 μL RPMI. After 24 h, the medium was removed and the cells were incubated for 3 days with RPMI in the presence or absence of various concentrations of extracts from *E. elatior* flowers. The concentration of extracts used ranged from 20, 40, 80, 160, 320, and 640 μg/mL. After incubation, 20 μL of MTT reagent was added to each well. The plate was then incubated in a CO_2_ incubator at 37 °C for 4 h. The reduced formazan products were quantified by measuring the absorbance at 570 nm using an ELISA reader. Each point represents the mean of triplicate experiments. The cell viability was determined using the formula:

2 $$ \mathsf{Viability}\ \left(\%\right) = \left(\mathsf{optical}\ \mathsf{d}\mathrm{e}\mathrm{n}\mathsf{sity}\ \mathsf{o}\mathsf{f}\ \mathsf{sampl}\mathrm{e}/\mathsf{optical}\ \mathsf{d}\mathrm{e}\mathrm{n}\mathsf{sity}\ \mathsf{o}\mathsf{f}\ \mathsf{c}\mathsf{o}\mathrm{n}\mathsf{trol}\right) \times \mathsf{100} $$

### Bacterial cultures and growth conditions

Multi drug resistant (MDR) clinical isolates of Gram-positive bacteria (*Staphylococcus aureus, Bacillus subtilis, Listeria monocytogenes*) and Gram-negative bacteria (*Escherichia coli, Salmonella typhimurium,* and *Pseudomonas aeruginosa*) with their antibiotic resistance profiles were obtained from the laboratory of microbial culture collection unit (UNiCC), Institute of Bioscience, Universiti Putra Malaysia, Selangor, Malaysia. All the test strains were maintained on nutrient agar slants at 4 °C and sub-cultured in nutrient broth for 24 h prior to testing. These bacteria served as test pathogens for the antibacterial activity assay.

### Antibacterial activity assay

Molten Mueller-Hinton agar, (15 mL at 45 °C, Oxoid, Basingstoke, UK) was poured into sterile Petri dishes (90 mm). Bacterial cell suspensions were prepared and 100 μL was spread evenly onto the surface of the agar plates. Once the plates had been aseptically dried, 6 mm wells were punched into the agar with a sterile Pasteur pipette. The test extracts (10 mg/mL) were dissolved in dimethylsulfoxide (DMSO)/water (1/9) and 80 μL were applied to the wells and incubated at 37 °C for 24 h. Gentamicin (25 μL/well at concentration of 4 μg/mL) and ciprofloxacin (5 μg/mL) were used as positive antibacterial controls. The antibacterial activity was evaluated by measuring the diameter of the circular inhibition zones around the well. Tests were performed in triplicate and values are the average of three replicates. Data are expressed as mean ± standard deviation.

### Minimum inhibitory concentration (MIC)

The minimum inhibitory concentration (MIC) of plant extracts was analyzed by the agar-well diffusion method with a protocol similar to that described in the previous section. A bacterial suspension (10^5^–10^6^ CFU/mL) of each tested microorganism was spread on the nutrient agar plate. Wells (6 mm diameter) were cut out of the agar, and 60 μL of each test extract at different concentrations (10, 20, 25, 50, 75 and 100 μg/mL) dissolved in DMSO, were applied to them. The plates were incubated at 37 °C for 24 h under aerobic conditions, followed by measurement of the diameter of the inhibition zone expressed in millimeter. The MIC was taken as the lowest concentration of test material where there was visually no growth after 24 h. All samples were tested in triplicate.

## Results and discussion

### Total phenolics, total flavonoids, and total tannin contents

Aqueous and ethanol extracts of *E. elatior* flowers collected from three different locations in Malaysia, were evaluated for phytochemical composition and antioxidant properties. As shown in Table 1, the flowers collected from different locations had significantly different concentrations of TPC and the values were also dependent on the solvent. Aqueous flower extracts from Kelantan exhibited the highest level of TPC (618.9 mg/100 g DM) compared with that of aqueous extracts of flowers collected from Pahang (544.7 mg/100 g DM) and Johor (516.4 mg/100 g DM). Extraction with water rather than ethanol enhanced the levels of TPC by about 15.9 % in extracts of flowers collected from Kelantan, 8.0 % from Pahang, and 10.6 % from Johor. Extracts of the *E. elatior* flowers had a higher TPC than that reported previously for other herbs including *Marrubium vulgare* (3.86 mg/100 g DM), *Rosmarinus officinalis* (1.71 mg/100 g DM), *Artemisia vulgaris* (3.83 mg/100 g DM), *Levisticum officinale* (0.72 mg/100 g DM), *Epilobium hirsutum* (4.03 mg/100 g DM), and *Chelidonium majus* (2.09 mg/100 g DM) [19].

**Table 1:** Total phenolic, total flavonoid and total tannins content of *E. elatior* flowers, extracted with different solvent and collected from three different locations

Locations	Solvents	TPC	TFC	TTC
Kelantan	Aqueous	618.9 ± 16.40^a^	354.2 ± 11.24^a^	122.5 ± 5.38^a^
	Ethanol	520.4 ± 15.26^c^	312.5 ± 10.44^c^	114.5 ± 5.72^b^
Pahang	Aqueous	544.7 ± 15.33^b^	330.8 ± 12.19^b^	106.4 ± 4.59^c^
	Ethanol	500.6 ± 14.54^d^	310.4 ± 11.72^c^	104.2 ± 4.68^c^
Johor	Aqueous	516.4 ± 15.41^c^	246.1 ± 12.80^d^	88.7 ± 3.50^d^
	Ethanol	461.5 ± 20.55^e^	211.0 ± 11.55^e^	86.3 ± 4.68^d^

All analyses are the mean of triplicate measurements ± standard deviation. Results expressed in mg/100 g DM. Means not sharing a common letter in each column were significantly different at *P* ≤ 0.05

**Table 2:** Antioxidant activity of *E. elatior* flowers, extracted with different solvent and collected from three different locations

Locations	Solvents	DPPH (%)	IC_50_ (μg/mL)	FRAP (mM Fe(II)/g)
Kelantan	Aqueous	76.4 ± 5.89^c^	34.5 ± 1.42^f^	6.88 ± 0.62^b^
	Ethanol	63.2 ± 4.31^e^	41.2 ± 2.16^e^	5.66 ± 0.75^c^
Pahang	Aqueous	70.5 ± 5.22^d^	44.6 ± 2.41^d^	6.11 ± 0.68^c^
	Ethanol	58.6 ± 3.81^f^	59.5 ± 3.17^b^	5.20 ± 0.70^c^
Johor	Aqueous	62.1 ± 4.79^e^	52.9 ± 2.88^c^	5.57 ± 0.63^c^
	Ethanol	40.0 ± 3.33^g^	139.8 ± 4.52^a^	4.06 ± 0.51^d^
BHT		92.0 ± 4.91^b^	19.7 ± 0.87^g^	6.05 ± 0.55^c^
α-tocopherol		98.6 ± 4.57^a^	12.6 ± 0.81^h^	7.78 ± 0.89^a^

All analyses are the mean of triplicate measurements ± standard deviation. Means not sharing a common letter in each column were significantly different at *P* ≤ 0.05

The amount of TFC extracted was between 211.0–354.2 mg/100 g DM and was significantly influenced by the different locations and solvents. The TFC (354.2 mg/100 g DM) was the highest in Kelantan extracts followed by Pahang (330.8 mg/g DM) and Johor (246.1 mg/g DM) samples. As for the TPC, aqueous extraction enhanced the level of TFC by about 11.7 % (Kelantan), 6.1 % (Pahang), and 14.2 % (Johor) compared to ethanol extraction. It is apparent from Table 1 that the solubility of polyphenolic compounds is higher in aqueous solvents than in ethanol. The TFC of extracts of *E. elatior* flowers from Kelantan was higher than that previously reported for herbs including *Cymbopogon citratus* (3.05 mg/g DM), *Mentha piperita* (3.01 mg/g DM), *Citrus bergamia* (2.11 mg/g DM), and *Jasminum officinale* (3.05 mg/g DM) [20]. Herbs may contain tannins, which are important phytochemicals with a wide range of medicinal properties, including anticancer, anti-inflammatory, antioxidant, and antibacterial activities [21–23]. Variable tannin content was identified in different herbs and plants including *Caesalpinia pyramidalis Tul.* (817 mg/100 g DM), *Anadenanthera colubrina (Vell.)* (4.41 mg/100 g DM), and *Jatropha mollissima* (2.35 mg/100 g DM) [24].

In the current study, *E. elatior* flowers from all locations had a high TTC. Aqueous extracts of *E. elatior* flowers from Kelantan had the highest TTC (122.5 mg/100 g DM) followed by Pahang (106.4 mg/100 g DM), and Johor samples (88.7 mg/100 g DM). The solvent did not appear to affect the TTC and no significant difference between the TTC of aqueous and ethanol extracts from Pahang and Johor was observed. Mailoa et al. [25] reported that the TTC in extracts of guava leaves decreased from 3.228 mg/g in ethanol 30 % to 2.33 mg/g in 70 % ethanol. A recent study showed that water was a more effective solvent than ethanol-water mixtures for the extraction of condensed tannins from grape skin [26].

### Antioxidant activity

**Fig. 2:**
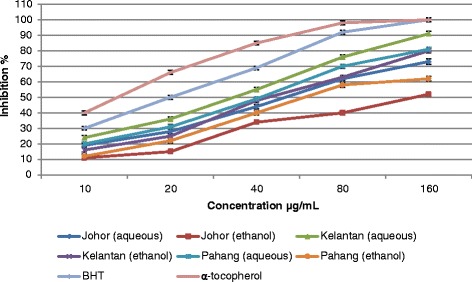
DPPH radical scavenging activity of *E. elatior* flowers, extracted with different solvent and collected from three different locations. Bars represent standard error of the means

The antioxidant properties of extracts of flowers of *E. elatior* from three different locations of Malaysia were determined using two different methods namely DPPH and FRAP assays. The results from both assays showed significant differences in the antioxidant activity between the different sampling locations and solvent type (Table 2), with aqueous extracts having greater DPPH and FRAP activity than the ethanol extracts. At a concentration of 100 μg/mL, the highest DPPH activity was observed in the aqueous extract of *E. elatior* flowers from Kelantan (76.4 %) followed by Pahang (70.5 %) and Johor sample extracts (62.1 %), with 50 % free radical scavenging (IC_50_) values of 34.5, 44.6, and 52.9 μg/mL, respectively compared to BHT (19.7 μg/mL) and α-tocopherol (12.6 μg/mL). The IC_50_ value increased by about 19.4 % (Kelantan), 33.4 % (Pahang), and 164.2 % (Johor), when the flowers were extracted with ethanol (Fig. 2). It should be noted that a lower IC_50_ value represents more potent free radical inhibition (strong free radical inhibitors are active at low concentrations). Thus, the results indicated that aqueous extracts have superior antioxidant activity compared to ethanolic extracts. Lachumy et al.[11] reported that methanol extracts of *E. elatior* flowers (0.031–2.000 mg/mL) showed anti-oxidant activity with a 2,2-diphenyl-1-picrylhydrazyl (DPPH) radical scavenging activity, IC_50_ of 9.14 μg/mL compared to 8.08 mg/mL with BHT. In another study, a methanol (50 %) extract of *E. elatior* flowers (8.33 mg/mL, 60 μL) showed anti-oxidant activity with Fe^2+^ reducing ability (3.6 mM Fe(II)/100 g) using ferric reducing antioxidant potential [7]. In a follow-up study, an ethanol (95 %) extract of fresh *E. elatior* flowers (50 μg/mL, 300 μL) showed anti-oxidant activity with Fe^2+^ reducing ability (930 mM Fe(II)/g fresh weight) using ferric reducing antioxidant potential [27].

**Table 3:** Identified phenolic acids and flavonoids from *E. elatior* extracts collected from three different locations

	Kelantan	Pahang	Johor
Phenolic acids			
Gallic acid	129.14 ± 7.54^a^	102.40 ± 9.07^b^	87.72 ± 6.74^c^
Tannic acid	82.66 ± 10.6^a^	66.19 ± 10.56^b^	53.70 ± 4.62^c^
Chlorogenic acid	75.79 ± 9.61^a^	70.45 ± 8.46^a^	ND
Caffeic acid	88.46 ± 7.20^a^	58.25 ± 4.56^c^	69.11 ± 4.02^b^
Flavonoids			
Quercetin	89.50 ± 6.55^a^	77.20 ± 7.80^b^	64.17 ± 5.66^c^
Apigenin	71.88 ± 7.19^a^	60.18 ± 5.06^b^	40.23 ± 5.21^c^
Kaempferol	62.19 ± 6.58^b^	70.28 ± 6.22^a^	55.70 ± 4.83^c^
Luteolin	46.69 ± 5.19^a^	ND	ND
Myricetin	35.75 ± 7.21^a^	20.58 ± 5.17^b^	5.66 ± 4.29^c^

All analyses are the mean of triplicate measurements ± standard deviation. Results expressed in mg/100 g DM. Means not sharing a common letter in each column were significantly different at *P* ≤ 0.05. ND: not detected

The FRAP value was in the range of 4.06–7.78 mM of Fe (II)/g with the highest and lowest FRAP activity observed in the aqueous extracts from Kelantan flowers and ethanol extracts from Johor flowers, respectively. The FRAP activity increased by about 17.7 % (Kelantan), 14.8 % (Pahang), and 37.1 % (Johor) when extraction was with aqueous solvent rather than ethanol. Chan et al. [4] reported that *Etlingera* species with high leaf TPC also have high antioxidant capacity and FRAP activity and several studies reported a significant correlation between the antioxidant activity of herbs and the phytochemical content [19, 28, 29]. In the current study, aqueous extracts of *E. elatior* flowers collected from Kelantan had the highest content of total flavonoids, total phenolics, and total tannins in addition to high antioxidant properties. Earlier, it has been opined that with the change of solvent polarity, viscosity, and vapor pressure, the type of antioxidant compound being dissolved in the solvent also varies. Solvents with low viscosity have low density and high diffusivity that allows them to easily diffuse into the pores of the plant materials to leach out the bioactive constituents [30].

### Separation and identification of phenolic acid and flavonoid compounds

**Fig. 3:**
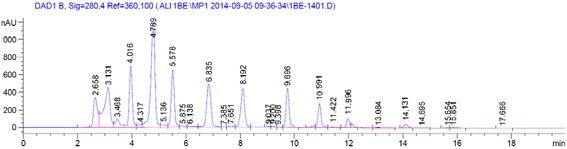
UHPLC full chromatogram of extract of *E. elatior* flowers extracts (Kelantan) showing peak corresponding to gallic acid (RT: 4.78 min), tannic acid (RT:6.83 min), chlorogenic acid (RT:8.19 min), caffeic acid (RT:5.57 min), quercetin (RT:4.016 min), apigenin (RT:9.69 min), kaempferol (RT:10.99 min), luteolin (RT:11.99 min) and myricetin (RT:14.13 min)

In the current study, four phenolic acids (gallic acid, tannic acid, chlorogenic acid, and caffeic acid) and five flavonoid compounds (quercetin, apigenin, kaempferol, luteolin, and myricetin) were separated and identified from the extracts of *E. elatior* flowers collected from three different locations (Table 3 and Fig. 3). An aqueous rather than an ethanolic extraction method was chosen for profiling of phenolic acids and flavonoids in order to maximize the TPC, TFC, TTC, and antioxidant activity. The results from the three different sampling locations showed significant differences. The highest content of gallic acid (129.14 mg/100 g DM), tannic acid (82.66 mg/100 g DM), chlorogenic acid (75.79 mg/100 g DM), and caffeic acid (88.46 mg/100 g DM) was observed in extracts of flowers collected from Kelantan. Chlorogenic acid was not detected in the extracts of flowers from Johor. The most important phenolic acids isolated in this study, based on concentration were gallic acid > caffeic acid > tannic acid > chlorogenic acid. The content of flavonoids was significantly different between the extracts of flowers from the three different locations. Compared with extracts of flowers from the other two locations, extracts collected from Kelantan had the highest content of quercetin (1.95 mg/g DM), rutin (1.48 mg/g DM), kaempferol (0.56 mg/g DM), catechin (89.5 mg/100 g DM), apigenin (71.88 mg/100 g DM), and myricetin (35.75 mg/100 g DM). Luteolin at a concentration of 48.69 mg/100 g DM was detected in extracts of flowers from Kelantan but not in the extracts from the other two locations.

Quercetin has been reported to be a potent antioxidant with anticancer activity [31, 32]. *E. elatior* flowers had higher levels of quercetin than other herbs such as *Silybum marianum* (23 mg/100 g DM), *Archangelica officinalis* (48 mg/100 g DM), and *Hypericum perforatum* (49 mg/100 g DM), but a lower quercetin content than *Chelidonium majus* (759 mg/100 g DM), *Epilobium hirsutum* (214 mg/100 g DM), *Juglans regia* (460 mg/100 g DM), and *Syzygium aromaticum* (155 mg/100 g DM) [19]. In addition, luteolin which has been reported to have potent anti- and pro-oxidative activity [31, 33, 34] was detected only in extracts of *E. elatior* from Kelantan where it was found in quantities higher than those reported for a number of other herbs such as *Salvia officinalis* (33.4 mg/100 g DM), *Poliomintha longiflora* (25.1 mg/100 g DM), and *Thymus vulgaris* (39.5 mg/100 g DM) [35]. The most important flavonoids isolated in this study, based on concentration were quercetin > apigenin > kaempferol > luteolin > myricetin. Comparing the three different sampling locations from the North-east (Kelantan) to South-east (Johor), the concentration of polyphenols decreased in the following order: Kelantan > Pahang > Johor. This variation in the content of phenolic acids and flavonoids in *E. elatior* flowers could be related to the differences in the weather conditions or soil nutrition and type, which have been reported previously [36–38]. This finding is in agreement with previous studies of current authors which found production and accumulation of secondary metabolites in herbs were influenced by growing area in Malaysia [39, 40].

### Anticancer activity

**Fig. 4:**
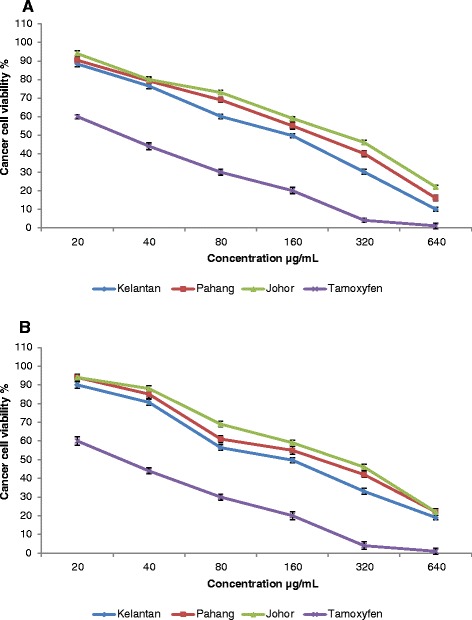
Anticancer activity of *E. elatior* flowers extracts (collected from three different locations) against MCF-7 (**a**) and MDA-MB-231 (**b**) cell lines. Bars represent standard error of the means

**Fig. 5:**
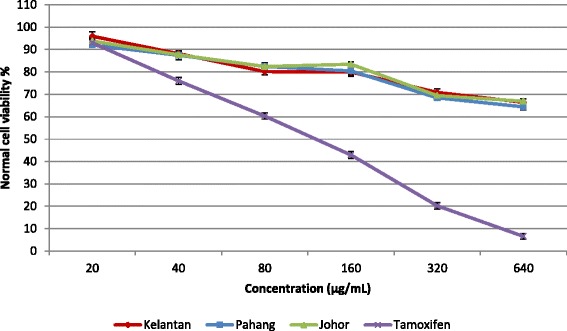
Toxicity effect of *E. elatior* flowers extracts (collected from three different locations) against normal cell line. Bars represent standard error of the means

Aqueous extracts of *E. elatior* flowers (20–640 μg/mL) collected from the three different locations (Kelantan, Pahang and Johor), were tested for anticancer properties against the human breast cancer cell lines, MCF-7 and MDA-MB-231 (Fig. 4). Extracts of flowers from Kelantan exhibited potent anticancer activity with IC_50_ of 173.1 and 196.2 μg/mL against MCF-7 and MDA-MB-231 respectively, compared to that of extracts of Pahang (IC_50_ = 204.5 and 246.2 μg/mL) and Johor samples (IC_50_ = 277.1 and 296.7 μg/mL). Tamoxifen as a positive control had IC_50_ values of 37.9 and 38.6 μg/mL against MCF-7 and MDA-MB-231 respectively. No toxic effect against normal cells was observed at concentrations of 20–640 μg/mL (Fig. 5), although tamoxifen was toxic against the normal cell line at concentrations above 120.4 μg/mL. Results of a recent study showed that ethanol extracts of *E. elatior* flowers (0–100 μg/mL) showed anti-tumor activity against MDA-MB-231 and MCF-7 (breast cancer) and HeLa (cervical cancer) with IC_50_ > 100 μg/mL [10]. Previous reports have described the anticancer activity of *E. elatior* against different cancer cell lines. Methanol extracts of *E. elatior* flowers (200 mg/mL) inhibited the Raji cell line by 85.9 % [9]. In addition, methanol (80 %) extracts of *E. elatior* flowers (0.1–100 mM) were cytotoxic against the MCF-7 cell line with an IC_50_ value of 47 μg/mL compared with tamoxifen with IC_50_ = 30 μM [9].

The anticancer properties of herbs and spices are directly related to their phytochemical content [41]. In the current study, the *E. elatior* extracts with the highest content of secondary metabolites exhibited the most potent antioxidant and anticancer activity. In general, therefore, it appears that the potent antioxidant and anticancer activity of *E. elatior* grown in the North-east of Malaysia may be attributed to the high phytochemical content.

### Antibacterial activity

An assessment of the antibacterial activity of *E. elatior* flower extracts collected from three different locations (Kelantan, Pahang and Johor) against both Gram-positive and Gram-negative bacteria is presented in Table 4. *E. elatior* flower extracts from Kelantan and Pahang demonstrated good antibacterial potential against both Gram-positive and -negative bacteria strains. As can be seen in Table 4, *E. elatior* extracts from Kelantan showed more potent antibacterial activity than flowers from Pahang with the exception of activity against *Staphylococcus aureus*. The antibacterial activity of *E. elatior* collected from Kelantan against *Staphylococcus aureus* (8.4  mm), *Bacillus subtilis* (6.5 mm) and *Pseudomonas aeruginosa* (8.0 mm) was higher than that of gentamicin (*Staphylococcus aureus* 6.5 mm; *Bacillus subtilis* 5.6 mm; *Pseudomonas aeruginosa* 6.5 mm) and ciprofloxacin (*Staphylococcus aureus* 7.3 mm; *Bacillus subtilis* 4.8 mm; *Pseudomonas aeruginosa* 6.7 mm). The most potent antibacterial activity of Kelantan extracts was observed against *Staphylococcus aureus* (8.4 mm) and *Pseudomonas aeruginosa* (8.0 mm). *Staphylococcus aureus* (9.2 mm) compared to other two locations which was higher than that of gentamicin (6.5mm) and ciprofloxacin (7.3mm). *E. elatior* flower extracts from Johor exhibited weak antibacterial activity compared with extracts from Kelantan and Pahang and did not show any antibacterial activity against *Listeria monocytogenes*, *Escherichia coli*, or *Pseudomonas aeruginosa* bacterial strains.

**Table 4:** Antibacterial activity of *E. elatior *flower extracts collected from different locations of Malaysia

Bacterial strains	Inhibition zone (mm)				
	Kelantan	Johor	Pahang	Gentamicin	Ciprofloxacin
*Staphylococcus aureus*	8.4 ± 0.264^b^	4.0 ± 0.142^e^	9.2 ± 0.316^a^	6.5 ± 0.277^d^	7.3 ± 0.276^c^
*Bacillus subtilis*	6.5 ± 0.216^a^	4.2 ± 0.207^e^	6.2 ± 0.168^b^	5.6 ± 0.264^c^	4.8 ± 0.229^d^
*Listeria monocytogenes*	2.5 ± 0.183^b^	NO	2.0 ± 0.273^c^	4.0 ± 0.177^a^	4.2 ± 0.119^a^
*Escherichia coli*	4.6 ± 0.166^b^	NO	2.6 ± 0.219^c^	5.4 ± 0.318^a^	5.5 ± 0.337^a^
*Salmonella typhimurium*	6.2 ± 0.250^c^	2.5 ± 0.266^e^	5.4 ± 0.348^d^	7.2 ± 0.372^a^	6.8 ± 0.352^b^
*Pseudomonas aeruginosa*	8.0 ± 0.233^a^	NO	6.1 ± 0.318^c^	6.5 ± 0.374^b^	6.7 ± 0.357^b^

All analyses are the mean of triplicate measurements ± standard deviation. Means not sharing a common letter in each row were significantly different at *P* ≤ 0.05. NO: not observed

The minimal inhibitory concentration (MIC) of *E. elatior* extracts ranged from 30 to > 100 μg/mL (Table 5). The lower value for MIC represents more potent antibacterial activity (strong bacterial inhibitors are active at low concentrations). The results showed that among the bacteria strains studied, *Staphylococcus aureus* is sensitive to *E. elatior* extracts from Pahang with a MIC value of 30.0 μg/mL and other bacterial strains are sensitive to Kelantan extracts with a MIC ranging from 40 to > 100 μg/mL. A recent study using the disk diffusion method showed that methanol (80 %) extracts of *E. elatior* flowers (100 mg/mL) inhibited the growth of *Staphylococcus aureus* with a MIC of 1.563 mg/mL, *Bacillus thuringienesis* (MIC = 6.250 mg/mL), *Escherichia coli* (MIC = 12.5 mg/mL), *Salmonella* sp (MIC = 12.5 mg/mL), *Microccocus* sp.(MIC = 50 mg/mL), *Bacillus subtilis* (MIC = 25 mg/mL), and *Proteus mirabilis* (MIC = 25 mg/mL) compared with chloramphenicol (30 μg/mL, *S. aureus* = 28 mg/mL, *B. thuringienesis* = 31 mg/mL, *E. coli* = 30 mg/mL, *Salmonella* sp. = 29 mg/mL, *Microccocus* sp. = 32 mg/mL, *B. subtilis* = 30 mg/mL, and *P. mirabilis* = 30 mg/mL) [11]. Another study using the disc diffusion assay showed that ethanol (100 %) extracts of *E. elatior* flowers (12.5 mg/mL) inhibited the growth of *Bacillus subtilis* with an inhibition zone diameter of 11.7 mm compared with gentamicin = 25.7 mm [27]. The results of the current study support the hypothesis that the antibacterial activity of plants depends on where it is grown. Several studies have demonstrated that the antibacterial activity of plants involves polyphenolic compounds especially flavonoids [42–44]. The current study is in agreement with this because *E. elatior *extracts with the highest content of phenolic compounds exhibited the most potent antibacterial activity [45–47].

**Table 5:** Minimal inhibitory concentration (MIC)of *E.elatior *flower extracts collected from different locations of Malaysia

Bacterial Strains	Kelantan	Johor	Pahang
*Staphylococcus aureus*	40.0	60.0	30.0
*Bacillus subtilis*	80.0	>100	>100
*Listeria monocytogenes*	40.0	NO	50.0
*Escherichia coli*	>100	NO	>100
*Salmonella typhimurium*	50.0	>100	>100
*Pseudomonas aeruginosa*	60.0	No	80.0

All analyses are the mean of triplicate measurements ± standard deviation. Results expressed in µg/ mL. NO: not observed

## Conclusion

This study demonstrated that aqueous solvents rather than ethanol are recommended for extraction of phenolic acids, flavonoids, and tannins from *E. elatior* flowers. The levels of secondary metabolites and the pharmaceutical quality of *E. elatior* flowers decreased from the South-east to North-east of Malaysia. In general, if the three different sampling locations from North-east (Kelantan) to South-east (Johor) are compared, the concentration of polyphenols, as well as the antioxidant, anticancer, and antibacterial activities decreased in the following order: Kelantan > Pahang > Johor. One of the most significant findings in this study is that the extracts of *E. elatior* flowers exhibited promising anticancer activity against the MCF-7 and MDA-MB-231 cancer cell lines. The extracts contained substantial amounts of effective phenolic and flavonoid compounds such as gallic acid, caffeic acid, quercetin, luteolin, and myricetin, which can inhibit the growth of breast cancer cell lines. In conclusion, these findings indicate that *E. elatior* flowers grown in the North-east of Malaysia (Kelantan) are a potential source of therapeutic compounds with antimicrobial activity and suggest areas for further investigation.
